# Assessment of the Current Knowledge and Practice of General Practitioners Towards Laryngopharyngeal Reflux in Saudi Arabia

**DOI:** 10.7759/cureus.38043

**Published:** 2023-04-24

**Authors:** Mujtaba Alrayah, Rajab Alzahrani, Mohammed A Alghamdi, Kholoud M Alghamdi, Faisal F Almutairi, Anwar A Alghamdi, Raghad A Alzahrani, Taif A Bajaber, Tahani F Alanazi, Haya A Alnafisah

**Affiliations:** 1 Unit of Otolaryngology, Department of Surgery, Faculty of Medicine, Al-Baha University, Al-Baha, SAU; 2 Department of Medicine, Faculty of Medicine, Al-Baha University, Al-Baha, SAU; 3 Department of Medicine, Unaizah College of Medicine and Medical Sciences, Qassim University, Unaizah, SAU; 4 Department of Medicine, Faculty of Medicine, Fakeeh College of Medical Sciences, Jeddah, SAU; 5 Department of Medicine, Faculty of Medicine, University of Tabuk, Tabuk, SAU; 6 Department of Medicine, Faculty of Medicine, Princess Nourah bint Abdulrahman University, Riyadh, SAU

**Keywords:** proton pump inhibitors (ppi), heartburn, gastroesophageal reflux disease, laryngopharyngeal reflux disease, general practitioners

## Abstract

Background

General practitioners (GPs) have a significant role in the diagnosis of patients with laryngopharyngeal reflux (LPR). Some published data revealed a lack of knowledge among GPs about the disease, consequently, this lack of knowledge impacted their performance. This survey aims to assess the current knowledge and practice of general practitioners regarding laryngopharyngeal reflux in Saudi Arabia.

Methodology

This survey study was conducted to assess the current knowledge and practice of general practitioners regarding laryngopharyngeal reflux in Saudi Arabia using an online questionnaire. The questionnaire was distributed and collected from the five regions in Saudi Arabia, which are The Central Region (Riyadh, Qassim), Eastern Region (Dammam, Al-Kharj, Al-Ahasa), Western Region (Makkah, Madinah, Jeddah), Southern Region (Asir, Najran, Jizan), and Northern Region (Tabuk, Jouf, Hail).

Results

In the current study, we collected data from 387 general practitioners, 61.8% of whom were aged between 21-30 years old, and 57.4% of the participants were males. Moreover, 40.6% of the participants thought that both LPR and gastroesophageal reflux disease (GERD) share pathophysiology, however, they are two different diseases considering their clinical presentation. Moreover, it was found that heartburn was the most known symptom of LPR among the participants (Mean score 2.14 (SD=1.31), where a lower score indicated more relation). Considering the treatment of LPR, 40.6% and 40.3% of the participants reported using proton pump inhibitors once or twice daily respectively. In contrast, antihistamine/H2 blockers, alginate, and magaldrate were used to a lesser extent as reported by 27.1%, 21.7%, and 12.1%.

Conclusion

The current study showed limited knowledge among general practitioners considering LPR with a higher rate of referring patients to other departments depending on symptoms which may increase the pressure on other departments of mild cases.

## Introduction

Laryngopharyngeal reflux (LPR) is the backflow of gastric contents into the laryngopharynx where it comes in contact with the mucous membrane of the upper aerodigestive tract [1,2]. Laryngopharyngeal reflux is a prevalent, not well-understood disease affecting a high proportion of patients who seek laryngology consultation [3,4].

The importance of LPR was acknowledged widely. However, the pathophysiology of LPR has not been understood completely [5] and the diagnostic criteria for LPR remain controversial [6,7]. Symptoms of laryngopharyngeal reflux include hoarseness, sore throat, throat-clearing, chronic cough, Globus sensation, dysphagia, and postnasal drip [7].

The basis for diagnosing this pathology is a combination of chronic or intermittent symptoms correlated with positive findings in the larynx [8]. A complete clinical history with an exhaustive otolaryngological physical examination is necessary for the identification of this disease [9]. The physical examination must consider changes in the oral mucosa, hyperemia of the posterior pharyngeal wall, and lingual tonsils. Anti-reflux medications as well as modification of daily lifestyle are critical to the management of reflux laryngitis [10]. Patients with suspected LPR are advised to avoid stimuli that aggravate acid reflux, such as drinking alcohol, smoking, fatty foods, chocolate, acidic foods, spicy foods, and caffeine [11,12].

General practitioners (GPs) have a significant role in diagnosing patients with LPR. Some published data revealed a lack of knowledge among GPs about the disease, consequently, this lack of knowledge impacted their performance [13]. This survey aims to assess the current knowledge and practice of general practitioners regarding laryngopharyngeal reflux in Saudi Arabia.

## Materials and methods

This is a cross-sectional study that was conducted in November 2022-March 2023 to assess the current knowledge and practice among general practitioners regarding laryngopharyngeal reflux in Saudi Arabia using an online questionnaire that was distributed through emails and social media applications.

This research study was approved by the Committee of Research Ethics at Al Baha University, Saudi Arabia (IRB number: REC/SUR//BU-FM/2022/65). Moreover, filling out the questionnaire was considered consent from the responders to share in the study.

The target population was the general practitioners working in Saudi Arabia. Inclusion criteria were all general practitioners working in Saudi Arabia while general practitioners who refused to participate in the study or with incomplete questionnaires were excluded from the study. The sample size was estimated with an online sample size calculator (Raosoft) using a margin of error of 5% and a confidence interval of 95%, assuming an average response for most of the questions of 50% and depending on an average number of general practitioners in Saudi Arabia which is 13453 according to Ministry of Health Statistical Yearbook 2021 [14]. The required sample for the study was 374 participants. A non-probability sampling technique was used to select participants depending on inclusion and exclusion criteria. Since the study targets general practitioners from all of Saudi Arabia's regions, we assigned data collectors from each region to facilitate the data collection process. The questionnaire was distributed and collected from the five regions in Saudi Arabia, which are The Central Region (Riyadh, Qassim), Eastern Region (Dammam, Al-Kharj, Al-Ahasa), Western Region (Makkah, Madinah, Jeddah), Southern Region (Asir, Najran, Jizan), and Northern Region (Tabuk, Jouf, Hail). We collected the responses from all these regions using the e-questionnaire.

The questionnaire consisted of 25 questions divided into six sections:

Section 1: Sociodemographic and occupation information (4 questions),

Section 2: Definition and epidemiology (2 questions),

Section 3: Clinical presentation (5 questions),

Section 4: Diagnostic approach (3 questions),

Section 5: Treatment (10 questions), and

Section 6: Skills (1 question).

Before being entered into the computer, the data that were collected were checked for accuracy and coded. For both the entering of data and the analysis of said data, the Statistical Package for Social Sciences (SPSS) Version 26 (IBM Corp., Armonk, NY) was utilized. For the descriptive statistics, we employed percentages, as well as mean, range, and standard deviation. The chi-square test was used to determine whether there is a correlation and difference between categorical variables. The Fisher exact test would be done whenever the situation called for it (if the frequency is less than 5 in one or more of the cells in contingency tables).

## Results

In the current study, we collected data from 387 general practitioners, 61.8% of whom were aged between 21-30 years while 30.5% were aged between 31-40 years. In addition, 57.4% of the participants were males while 71.3% of them reported having 1-5 years of experience and 84.2% were working in governmental institutions (Table 1).

**Table 1:** Demographic factors of the participants (N=387).

		Count	N %
Age group	21-30	239	61.8%
	31-40	118	30.5%
	41-50	24	6.2%
	51-60	5	1.3%
	> 60	1	0.3%
Gender	Male	222	57.4%
	Female	165	42.6%
Number of years in practice	1-5	276	71.3%
	6-10	79	20.4%
	10-20	22	5.7%
	> 20	10	2.6%
Place of practice	Governmental	326	84.2%
	Private	61	15.8%

Moreover, 40.6% of the participants thought that both laryngopharyngeal reflux (LPR) and gastroesophageal reflux disease (GERD) share pathophysiology, however, they are two different diseases considering their clinical presentation while 29.5% of them thought that they are two different diseases regarding pathophysiological mechanisms, and 17.6% of them reported that they did not know whether there is a relationship between LPR and GERD. In addition, we found that 33.6% of the participants reported that they did not know the incidence of LPR in outpatients consulting the general consultation of the ENT department while 26.1% of the participants reported an incidence of 11-50% and 13.7% of between 6-10%. In addition, chronic cough throat was the most known condition associated with LPR as reported by 57.8% of the participants followed by the recurrent sore throat (47.9%), chronic voice disorders (40.2%), bronchial hypersensitivity (39.6%) and laryngotracheal stenosis (38.3%). On the other hand, only less than one-fifth of the sample related between each type of otitis media and LPR (Table 2).

**Table 2:** The knowledge considering definitions and epidemiology of LPR among the participants.

		Count	N %
What do you think about the relationship between laryngopharyngeal reflux (LPR) and gastroesophageal reflux disease (GERD)?	They are two different diseases regarding pathophysiological mechanisms.	114	29.5%
	They share pathophysiology, but are two different diseases regarding the clinical presentation	157	40.6%
	LPR is an unusual manifestation of GERD.	48	12.4%
	I don’t know.	68	17.6%
According to your experience, what’s the approximate incidence of LPR in outpatients consulting the general consultation of the ENT department?	I don’t know	130	33.6%
	0-2%	46	11.9%
	3-5%	24	6.2%
	6-10%	53	13.7%
	11-50%	101	26.1%
	> 50%	33	8.5%
Which of the following conditions are associated with LPR? (Several possible answers)	Bronchial hypersensitivity	153	39.6%
	Chronic rhinosinusitis	130	33.7%
	Acute otitis media	73	18.9%
	Chronic otitis media	80	20.7%
	Eustachian tube dysfunction	89	23.1%
	Reinke’s edema of the vocal folds (polypoid cordites)	97	25.1%
	Vocal fold nodules	133	34.5%
	Laryngotracheal stenosis	148	38.3%
	Recurrent sore throat	185	47.9%
	Chronic nasal obstruction	98	25.4%
	Chronic cough	223	57.8%
	Chronic voice disorders	155	40.2%

**Figure 1:**
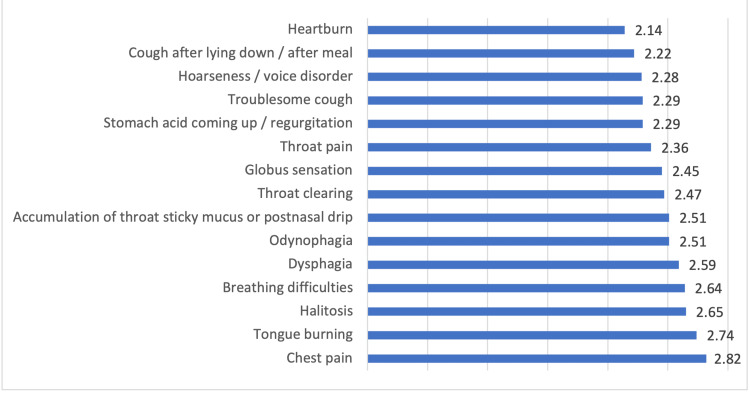
Known symptoms of laryngopharyngeal reflux (LPR) among the participants

Moreover, it was found that heartburn was the most commonly known symptom of LPR among the participants (Mean score 2.14 (SD=1.31), where lower score indicated more relation) followed by cough after lying down/after meals (2.22, SD=1.16), hoarseness/voice disorder (2.28, SD=1.19), troublesome cough (2.29, SD=1.15), and stomach acid coming up (2.29, SD=1.25). On the other hand, chest pain, tongue burning, halitosis, and breathing difficulties were the least known symptoms associated with LPR (Figure 1).

**Figure 2:**
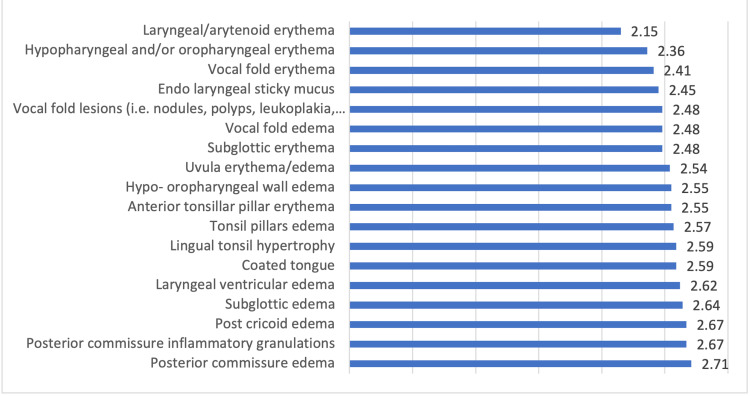
Signs of laryngopharyngeal reflux (LPR) known by the participants

Considering signs, laryngeal/arytenoid erythema was the most known sign related to LPR followed by hypopharyngeal and/or oropharyngeal erythema, vocal fold erythema, and endolaryngeal sticky mucus while posterior commissure edema was the least known symptoms by the participants (Figure 2).

Moreover, we found that 21.2% of the participants reported using some clinical tools as a questionnaire to assess the LPR symptoms or signs in their routine patient care while 29.2% reported sometimes using them and 49.6% reported not using them. In addition, 24.0% of the participants reported that 20-30% of the patients with LPR would come up with symptoms of heartburn while 21.7% thought that the prevalence is 30-40%, 16.0% of participants reported less than 20% and the rest of the participants thought that it is more than 40%. Considering the use of diagnostic tools, we found that 30.7% of the participants reported never using these tools and only depending on symptoms of LPR to make the diagnosis while 26.4% depended only on symptoms and signs of LPR and 31.3% depended on symptoms, signs, and positive response to an empirical therapeutic trial to confirm the diagnosis. In addition, the most used tools as a first tool for diagnosis included esophagogastroduodenoscopy (20.4%), esophageal manometry (16.3%), dual probe pH-monitoring (esophageal and pharyngeal probes) (16%) and oropharyngeal pH-monitoring (16.0%) while 30.2% of the participants would refer the patient to the gastroenterology department as the first option (Table 3).

**Table 3:** Diagnostic tests obtained by the participants to validate or investigate the diagnosis of laryngopharyngeal reflux.

Which of the following adjunctive diagnostic tests do you most commonly obtain to further validate or investigate your diagnosis of laryngopharyngeal reflux?												
	1	2	3	4	5	6	7	8	9	10	11	12
None. I only consider symptoms of LPR to make the diagnosis	30.7%	23.5%	14.2%	4.4%	5.2%	3.9%	2.6%	1.3%	1.6%	2.3%	2.6%	7.8%
None. I only consider symptoms & signs of LPR to make the diagnosis	26.4%	33.1%	12.1%	5.7%	5.2%	2.8%	3.4%	1.8%	0.8%	1.8%	2.3%	4.7%
None. I consider symptoms & signs and positive response to an empirical therapeutic trial to confirm the diagnosis.	31.3%	25.6%	15.8%	6.7%	3.9%	3.4%	3.1%	0.5%	2.3%	1.6%	1.3%	4.7%
Esophagogastroduodenoscopy	20.4%	21.4%	16.8%	7.5%	6.2%	5.7%	3.1%	2.1%	1.0%	2.1%	2.3%	11.4%
Trans nasal esophagoscopy	15.0%	22.0%	17.8%	8.3%	6.5%	4.7%	4.7%	2.8%	2.8%	1.8%	2.8%	10.9%
Esophageal manometry	16.3%	23.0%	16.5%	9.3%	5.2%	4.7%	5.9%	2.3%	2.1%	0.8%	3.1%	10.9%
Single probe pH-monitoring	15.2%	20.4%	20.4%	10.1%	5.7%	5.2%	5.2%	1.8%	2.3%	1.3%	3.4%	9.0%
Dual probe pH-monitoring (esophageal & pharyngeal probes)	16.0%	20.7%	17.1%	7.5%	7.2%	6.7%	4.1%	3.4%	1.8%	1.6%	3.1%	10.9%
Dual probe pH-monitoring (proximal and distal esophageal probes)	14.5%	22.5%	16.0%	7.2%	7.2%	5.2%	5.4%	2.1%	2.3%	2.3%	3.1%	12.1%
Intraluminal multichannel pH-impedance monitoring	14.7%	18.6%	16.3%	8.5%	6.5%	6.7%	4.9%	4.4%	1.3%	3.1%	3.9%	11.1%
Oropharyngeal pH-monitoring (Restech)	16.0%	19.1%	15.0%	9.0%	6.7%	5.2%	4.1%	3.1%	2.3%	2.8%	5.7%	10.9%
Pepsin detection in sputum (Peptest)	14.0%	20.7%	12.4%	9.3%	5.7%	5.7%	6.2%	2.6%	2.6%	2.8%	2.8%	15.2%
I refer the patient to the gastroenterology department	30.2%	19.4%	16.3%	7.5%	5.7%	3.4%	4.1%	2.6%	1.6%	2.1%	1.8%	5.4%

In addition, 15.2% of the participants reported that all patients must have a GI endoscopy while 45.5% of them thought that all patients with reflux disease refractory to medical management must have a GI endoscopy, 32.8% of them thought that GI endoscopy should be conducted among all elderly patients, and 31.3% thought that GI endoscopy should be conducted among patients whose symptoms require long-term proton pump inhibitors (PPI) (Table 4).

**Table 4:** The place of gastrointestinal (GI) endoscopy in the management of LPR known by the participants.

What’s the place of gastrointestinal (GI) endoscopy in the management of LPR? (select one or several responses)			
		Percent of Cases	
		N	
What’s the place of gastrointestinal (GI) endoscopy in the management of LPR? (select one or several responses)	All patients must have GI endoscopy	59	15.2%
	All patients with heartburn or stomach acid coming up must have a GI endoscopy	100	25.8%
	All elderly patients must have a GI endoscopy	127	32.8%
	All patients with reflux disease refractory to medical management must have a GI endoscopy	176	45.5%
	Patients whose symptoms require long-term PPI must have a GI endoscopy	121	31.3%
	I think that GI endoscopy is not important for LPR	46	11.9%
	I don’t know	81	20.9%

**Figure 3:**
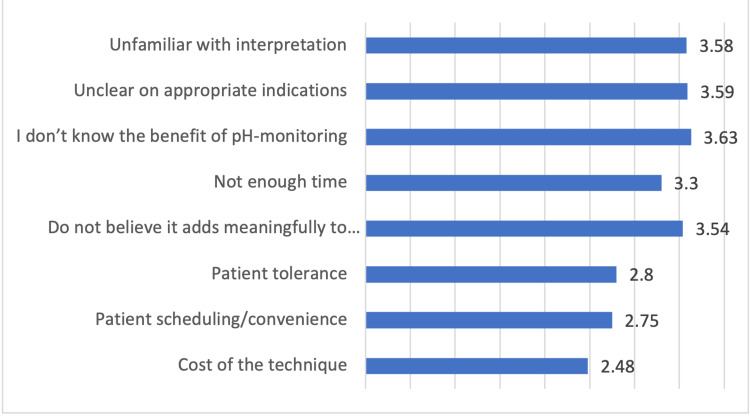
Barriers against the use of pH-monitoring testing by the participants

The limited knowledge about the importance of pH monitoring among physicians was the most common barrier against the use of pH-monitoring testing as part of their practice followed by unclear knowledge about the appropriate indications, being unfamiliar with interpretation, and thinking that it does not add meaning to patients care (Figure 3).

Considering the treatment of LPR, 40.6% and 40.3% of the participants reported using proton pump inhibitors once or twice daily respectively while antihistamine/H2 blockers, alginate, and magaldrate were used to a lesser extent as reported by 27.1%, 21.7%, and 12.1%. However, 22.5% of the participants reported that they refer all patients to the gastroenterology department without any treatment. Moreover, 83.5% of the participants reported that they systematically prescribe diet and behavioral changes for the treatment and 42.1% would prescribe medications for four weeks before reevaluating the cases. In addition, 46.6% of the participants would evaluate the improvement depending on both the symptom and finding improvements. Moreover, most of the participants reported that more than 31% of the patients respond to treatment. In case of failure of the treatment, 34.1% of the participants would refer patients to the gastroenterology department while 26.1% would make an additional examination. To treat non-acid biliary reflux, 31.3% of the participants would start with proton pump inhibitors. In addition, for patients with mild LPR, 39.8% of the participants would describe diet, behavioral changes, and proton pump inhibitors. Moreover, 29.7% of the participants thought that they are adequately knowledgeable and skilled in LPR (Table 5).

**Table 5:** The knowledge of the participants toward treatment strategies for LPR.

		Count	N %
What are the drugs that you usually use for the treatment of presumed LPR?	Proton pump inhibitors once daily	156	40.3%
	Proton pump inhibitors twice daily	157	40.6%
	Alginate (Gaviscon®, etc.)	84	21.7%
	Magaldrate (Riopan®, etc.)	47	12.1%
	Antihistamine / H2 blocker (Ranitidine®, etc.)	105	27.1%
	I don’t know because I refer the patient to the gastroenterology department.	87	22.5%
In practice, do you systematically prescribe diet and behavioral changes for the treatment?	No	64	16.5%
	Yes	323	83.5%
How long do you initially treat your patients in order to evaluate for response?	4 weeks	163	42.1%
	5-8 weeks	98	25.3%
	2-3 months	67	17.3%
	4 months	20	5.2%
	6 months	22	5.7%
	>6 months	8	2.1%
	Referring cases	9	2.3%
What’s the most important clinical outcome for the therapeutic response?	Symptom improvement	145	37.8%
	Findings improvement	49	12.8%
	Both symptoms and finding improvements	179	46.6%
	Improvement of pH-monitoring findings	11	2.9%
What is your impression of the % of patients who respond to treatment?	Not know	74	19.1%
	0-10%	10	2.6%
	11-30%	26	6.7%
	31-50%	78	20.2%
	51-70%	92	23.8%
	71-100%	107	27.6%
What do you make after the therapeutic period if some/a few symptoms persist?	I prescribe medication for a long period	65	16.8%
	I make additional examination	101	26.1%
	I just prescribe diet & behavioral changes	64	16.5%
	I refer the patient to the gastroenterology department	132	34.1%
	I refer the patient to the digestive surgery department for fundoplication	25	6.5%
According to your experience, what is the treatment of biliary (non-acid) reflux?	I do not know	33	8.5%
	Proton pump inhibitors (PPIs)	121	31.3%
	Alginate (Gaviscon®)	18	4.7%
	Magaldrate (Riopan®, etc.)	28	7.2%
	Association between PPIs, Alginate (Gaviscon®, etc.), Magaldrate (Riopan®, etc.)	74	19.1%
	Surgery (fundoplication)	71	18.3%
	Strict diet	42	10.9%
For patients with mild LPR, what do you prescribe?	Diet and behavioral changes	126	32.6%
	Proton pump inhibitors	72	18.6%
	Diet, behavioral changes, and proton pump inhibitors	154	39.8%
	Other medical treatment	11	2.8%
	Nothing	24	6.2%
What’s the most important factor explaining the resistance to treatment?	Biliary reflux	49	12.7%
	Lack of compliance of the patient	96	24.8%
	The severity of the reflux	66	17.1%
	The poor dietary habits and lifestyle of the patient	103	26.6%
	I don’t know	73	18.9%
Do you think that you are adequately knowledgeable and skilled about LPR?	No	155	40.1%
	Yes	115	29.7%
	I do not know	117	30.2%

In addition, it was found that general practitioners in age groups 31-40 and 41-50 years reported being more adequately knowledgeable and skilled about LPR than other age groups significantly (P=0.000). No significant difference was found between the two genders (P=0.365), however, the male seems to be slightly higher knowledgeable and skilled about LPR. Moreover, the knowledge increases with the increase in the years of experience significantly (P=0.030). No significant difference between physicians of governmental and private hospitals considering knowledge (P=0.790) (Table 6).

**Table 6:** The relation between demographic factors and knowledge about LPR.

		Do you think that you are adequately knowledgeable and skilled about LPR?				
		No		Yes		
		Count	N %	Count	N %	
Age group	21-30	187	78.2%	52	21.8%	0.000*
	31-40	67	56.8%	51	43.2%	
	41-50	13	54.2%	11	45.8%	
	51-60	4	80.0%	1	20.0%	
	> 60	1	100.0%	0	0.0%	
Gender	Male	152	68.5%	70	31.5%	0.365
	Female	120	72.7%	45	27.3%	
Number of years in practice	1-5	206	74.6%	70	25.4%	0.030*
	6-10	46	58.2%	33	41.8%	
	10-20	14	63.6%	8	36.4%	
	> 20	6	60.0%	4	40.0%	
Place of practice	Governmental	230	70.6%	96	29.4%	0.790
	Private	42	68.9%	19	31.1%	

## Discussion

Since Koufman first comprehensively detailed the symptoms of LPR in 1991, the condition has been acknowledged as a distinct disease [15]. Over the course of the previous few decades, there has been a steady rise in the total number of publications about LPR [16]. The diagnosis and treatment of LPR have been more standardized in recent years thanks to the availability of both scientific and clinical evidence. In most cases, the first step for people who encounter symptoms of LPR is to make an appointment with their family doctor or general practitioner (GP). It has been observed that general practitioners in the UK have a limited understanding of LPR [17]. Even if a treatment protocol has been proposed to help general practitioners manage patients with LPR [18], most GPs will still refer their patients to specialist facilities for additional evaluation and counseling. The purpose of this study was to evaluate the knowledge and practices of Saudi Arabian general practitioners about laryngopharyngeal reflux disease.

In the present study, it was discovered that 29.7% of the participants believe that they have sufficient knowledge and skills regarding LPR. This conclusion lends credence to the findings of several earlier research that general practitioners had a relatively low level of knowledge. In a local study that evaluated the LPR management practices of residents and consultants in three government hospitals in Riyadh, the researchers discovered that 41.3% of the residents and 27.8% of the consultants were unaware of any recommendations for the administration of patients with LPR [19]. Few studies have been conducted on a global scale to evaluate the LPR management expertise of family doctors, and these studies have not focused on residents [20,21]. The most recent data demonstrated that older residents and those training in internal medicine had a higher degree of expertise than those training in family medicine who were younger. In the management of LPR, it has been demonstrated that one's age and specialty can be used to predict awareness and prescription behavior [22]. There is a correlation between age and increased exposure to various knowledge sources and training [19]. Specialists such as gastroenterologists and internists are more likely to be aware of and follow the LPR's standard management guidelines better. As a result, they have a larger possibility of experiencing better outcomes [20-22]. This conclusion demonstrates the critical requirement for educational programs for residents in the fields of family and internal medicine. The fact that roughly 60% of these people viewed their level of LPR management competence as being somewhere between partially competent and non-competent may imply a favorable reception of the proposed instructional sessions. It was discovered that primary care physicians' understanding and adherence to LPR management could be considerably improved through participation in multifaceted continuing medical education courses. These courses include both lectures and practical discussions with senior personnel [23,24]. Improved knowledge was associated with better referral practices and better detection of unusual presentations of LPR in this study, even though this was not always the case. The study was conducted in the United Kingdom.

The absence of typical symptoms and signs associated with gastroesophageal reflux disease (GERD) on endoscopy [18] leads to a high rate of incorrect diagnosis of LPR in basic care settings. In patients with LPR, GERD has been hypothesized to be an underlying cause [25]. Therefore, both conditions share the same etiology despite having distinct symptoms; however, only 40.6% of the people who took part in the current study were aware that LPR and GERD, despite having similar pathophysiology, are two distinct diseases due to the differences in how they manifest themselves clinically.

In addition, 57.8% of the participants reported having chronic cough throat as the most known condition associated with LPR. This was followed by recurrent sore throat (47.9%), chronic voice disorders (40.2%), bronchial hypersensitivity (39.6%), and laryngotracheal stenosis (38.3%). Laryngopharyngeal reflux, also known as LPR, is an extraesophageal form of gastroesophageal reflux disease. It is characterized by a persistent cough, hoarseness, dysphonia, frequent throat clearing, and globus pharyngeus. Laryngopharyngeal reflux can be treated with medication [26]. It is estimated that LPR is responsible for 10% of all patients seen in ENT clinics and accounts for 50% of patients with voice issues [15]. However, because there is no testing that is the gold standard, the incidence of LPR may be overestimated. For example, one meta-analysis that looked at the data from pH probe readings reported that anywhere from 10 to 60% of normal participants had reflux [27,28]. Moreover, 33.6% of the participants in the current study said that they did not know the incidence of LPR in outpatients consulting the general consultation of the ENT department. In contrast, 26.1% of the participants reported an incidence of 11-50%, and 13.7% of the participants reported an incidence of between 6-10%.

In the current study, many participants described heartburn as the most known symptom of LPR. This indicates a misunderstanding of the difference between the symptoms of LPR and GERD, in which symptoms such as heartburn, regurgitation, and supine (nocturnal) reflux were significantly associated with GERD more than LPR [29]. However, the people in our sample were able to identify other symptoms, such as a cough that occurred after lying down or after eating, hoarseness or voice issue, and a cough that was difficult to control.

It might be difficult to assess and diagnose LPR because different people experience different symptoms. There have been several different ideas put forward for assessment tools, however, none of these methods are completely reliable. In the current study, 21.2% of the participants reported using clinical tools, such as questionnaires, to assess the LPR symptoms or signs in their routine patient care. Meanwhile, 29.2% of the participants reported using these clinical tools sometimes, and 49.6% of the participants said not using these clinical tools at all. In addition, esophagogastroduodenoscopy (20.4%), esophageal manometry (16.3%), dual probe pH-monitoring (esophageal & pharyngeal probes) (16%), and oropharyngeal pH-monitoring (16.0%) were the most common tools used as the first tool for diagnosis. However, 30.2% of the participants would refer the patient to the gastroenterology department as the first option. Monitoring of 24-hour multichannel intraluminal impedance-pH (MII-pH) is now regarded as the most reliable approach for determining the existence of LPR. In this diagnostic procedure, a probe is used to look for the presence of liquid and/or gas reflux into the esophagus and throat. It gives a profile of the reflux that includes the kind of reflux (acid, weakly acid, or non-acid reflux), the frequency of the reflux, and the timing of the reflux (daytime or nighttime). However, because this is only a snapshot of a person's presentation over the course of 24 hours, it may not be reflective of their LPR picture. This is because LPR can frequently come in spurts [30]. However, in the current study, we found that most of the participants had limited knowledge about the significance of pH monitoring, were unfamiliar with interpretation, or had unclear knowledge about the appropriate indications. This results in a reduction in the number of applications that can be made of such a tool in the present circumstance. Endoscopy, barium studies, and pH probes are the tests that are requested the most frequently by GERD patients, according to previous local [19] and international [20,21] research, which demonstrated a considerably lower utilization of testing overall. However, the high propensity of general practitioners to refer patients to the gastroenterology department as the first option without initiating evaluation or treatment could increase the pressure on these departments, resulting in a decrease in the quality of healthcare services provided to patients.

In individuals suspected of having LPR, empiric therapy consisting of twice-daily PPI administration is now regarded as the most effective diagnostic and therapeutic test. When it came to the treatment of LPR, 40.6% and 40.3% of the participants said that they used proton pump inhibitors (PPIs) once or twice a day, respectively. PPIs are nevertheless widely recommended by referring ENT doctors, even though research has provided no support for the idea that using PPIs is more effective than using a placebo. Although the uncontrolled trials reported positive results, the randomized controlled trials (RCTs) did not show any difference in symptom response to empiric PPI treatment for LPR, according to the findings of a systematic review that included 14 uncontrolled studies and six RCTs that were controlled with a placebo [31]. PPI medication was found to have a moderate but nonsignificant therapeutic advantage over placebo in a meta-analysis that pooled the data from eight RCTs that involved a total of 344 patients who had suspected GERD-related persistent laryngitis (relative risk, 1.28; 95% CI, 0.94-1.74) [32]. The most recent meta-analysis, which included 14 RCTs with a total of 771 participants, concluded that patients treated with PPI medication had a considerably higher response rate in comparison to those who got a placebo (risk difference, 0.15; 95% CI, 0.01-0.30) [33]. In addition, diet, behavioral adjustments, and proton pump inhibitors were described by 39.8% of the individuals who had patients with mild LPR. There is a general agreement among researchers that food and lifestyle recommendations are necessary for the effective long-term management of LPR [34,35]. In a recent study, foods, and fluids that are regularly consumed in Europe were categorized according to their reflexogenic potential to provide patients with LPR with information regarding products that they should avoid [30]. According to the findings of one study, the effects of PPI and an "alkaline" Mediterranean diet based on plant proteins, cooked vegetables, and low levels of animal fats on the symptoms of LPR were found to be comparable. The "alkaline" Mediterranean diet consisted of plant proteins, cooked vegetables, and low levels of animal fats. It has been hypothesized that dietary changes alone may be sufficient to treat most cases of mild to moderate LPR, hence reducing the need for medical care as well as the risk of unneeded pharmaceutical exposure [36].

The limitation of our study is that it was restricted to the GPs practicing in big cities of Saudi Arabia. Thus, it is not representative of GPs in other cities of the country, especially remote rural areas. A broader national survey would be needed to test the generalizability of our results. Added to that, this was a cross-sectional survey of self-reported knowledge and self-reporting physicians may underestimate their knowledge in an area. The nuances of clinically complex areas such as this are difficult to fully address with a single study that relies primarily on self-reporting measures. However, since there is very little research exploring GPs' experiences towards LPR in Saudi Arabia our chosen methodological approach is a necessary and reasonable place to start.

## Conclusions

In conclusion, the current study showed that there is insufficient knowledge among general practitioners concerning LPR with a higher rate of referring patients to other departments depending on only symptoms which may increase the pressure on these departments for mild cases. Therefore, it is important to increase the knowledge of general practitioners regarding LPR.
